# Symptom, Functional, and Work Participation Profiles Among Racialized Canadians with Pre-Existing Mental Health Challenges and Long COVID: A Cross-Sectional Study

**DOI:** 10.3390/healthcare14121726

**Published:** 2026-06-16

**Authors:** Maryam Shahzad, Sana Siddiqui, Chloe Lau, De-Lawrence Lamptey, Victor E. Ezeugwu, Geoffrey Maina, Chris J. Maddison, Kimberly Flowers, Armaan Rehman Shah, Thinuri Welithotage, Behdin Nowrouzi-Kia

**Affiliations:** 1 Rehabilitation Sciences Institute, Temerty Faculty of Medicine, University of Toronto, Toronto, ON M5G 1V, Canada; 2 Department of Occupational Science and Occupational Therapy, Temerty Faculty of Medicine, University of Toronto, Toronto, ON M5S 2E4, Canada; 3 Bloorview Research Institute, Holland Bloorview Kids Rehabilitation Hospital, Toronto, ON M4G 1R8, Canada; 4 Department of Physical Therapy, Faculty of Rehabilitation Medicine, University of Alberta, Edmonton, AB T6G 2G4, Canada; 5 College of Nursing, Prince Albert Campus, University of Saskatchewan, 130-1061 Central Avenue, Prince, AB S6V 4W4, Canada; 6 Department of Computer Science, University of Toronto, Toronto, ON M5S 2E4, Canada; 7 Independent Researcher, Edmonton, AB T5T 2W8, Canada; kclouson@hotmail.com; 8 Krembil Research Institute, University Health Network, Toronto, ON M5T 0S8, Canada; 9 Institute for Mental Health Policy Research, Centre for Addiction and Mental Health, 250 College St., Toronto, ON M5T 1R8, Canada

**Keywords:** long COVID, functioning, symptoms, mental health, Canada

## Abstract

**Highlights:**

**What are the main findings?**

- Racialized individuals with pre-existing mental health conditions and long COVID reported moderate functional impairment alongside clinically significant fatigue and post-exertional malaise.
- Symptom burden was highest in respiratory and psychological domains, with notable cognitive difficulties and mild-to-moderate anxiety and depression.

**What are the implications of the main findings?**

- Long COVID in populations with intersecting vulnerabilities may be approached as a syndemic condition requiring integrated, multidisciplinary care.
- Rehabilitation and public health strategies should incorporate equity-informed approaches that address both clinical symptoms and social determinants of health.

**Abstract:**

Background/objectives: Long COVID is associated with persistent, multi-system symptoms, yet little is known about how it affects individuals with intersecting vulnerabilities, such as a racialized identity and pre-existing mental health conditions. This study aimed to descriptively characterize the symptom burden, functional outcomes and mental health in this population. Methods: A cross-sectional, exploratory study was conducted among 51 adults in Canada who self-identified as racialized and as having a pre-existing mental health condition and reported long COVID symptoms. Participants completed an online survey, including validated measures of symptoms, fatigue, post-exertional malaise, cognitive function, mental health and disability. Descriptive statistics were used to summarize outcomes. Results: Participants reported a slight to moderate overall symptom burden, with the highest scores in respiratory and psychological domains. Functional impairment was moderate across work, social and daily activities (Work and Social Adjustment Scale mean = 17.35; World Health Organization Disability Assessment Schedule 2.0 mean = 16.61; Post COVID-19 Functional Status Scale mean = 2.20). Fatigue and post-exertional malaise were notable (Modified Fatigue Impact Scale mean = 43.39; DePaul Symptom Questionnaire—Post-Exertional Malaise mean = 22.47), and cognitive difficulties were commonly reported (Perceived Deficits Questionnaire mean = 33.43). Anxiety and depression scores were in the mild to moderate range respectively (General Anxiety Disorder-7 mean = 9.27; Patient Health Questionnaire-9 mean = 11.43). Conclusions: Clinically relevant fatigue, post-exertional malaise, and depression were found, alongside moderate functional limitations across life domains. The findings support the conceptualization of long COVID as a syndemic condition and underscore the need for equity-informed research, rehabilitation and public health strategies.

## 1. Introduction

Long COVID has been defined as the presence of persistent, new, or recurrent symptoms that continue for weeks to years following the initial infection with SARS-CoV-2, the virus that causes COVID-19 [1]. Symptoms are wide-ranging and can involve several organ systems, including respiratory, neuro-cognitive, musculoskeletal, and gastrointestinal. Most commonly, individuals may experience shortness of breath, brain fog, muscle pain and weakness, and digestive issues such as chronic diarrhea [1]. Prevalence estimates indicate that 5% to 30% of people who contract COVID-19 may develop long COVID, although rates vary across studies and populations [1]. Risk factors for developing long COVID may include severe COVID-19 requiring hospitalization, the presence of pre-existing conditions (e.g., asthma), women, and those with repeat infection [2].

Beyond disease processes, increasingly, research has provided support for the conceptualization of long COVID as a syndemic condition, characterized by the reinforcing interaction between multiple health conditions and systemic inequities that shape the burden of COVID-19 and long COVID [3]. Much of the existing syndemic literature has emerged from the Global South and low- and middle-income countries, where data has emphasized the interaction between chronic illness and mental health in marginalized populations. In the North American context [4], higher mortality rates have been observed among racialized and marginalized groups, with an elevated risk reported among African American and Hispanic/Latino communities in the United States [5]. A systematic review and meta-analysis identified that compared to white people, Black people faced a significantly greater burden of COVID-19, including a higher prevalence, hospitalization and mortality [6]. These inequities extend into Long COVID as well, with a higher prevalence among Black and Hispanic populations, as well as among females [7]. Emerging Canadian evidence further underscores how systemic factors shaped COVID-19-related health outcomes. A national cross-sectional study using Statistics Canada’s crowdsourcing survey (*n* = 32,605) found that cumulative exposure to intersectional discrimination in the two years prior to, and during, the pandemic was disproportionately experienced by racialized minorities and Indigenous people. Importantly, a dose–response relationship was observed between cumulative exposure to discrimination and long-term health conditions such as long COVID [8]. Explicitly, greater cumulative exposure to discrimination (e.g., experiencing more discrimination) was associated with progressively higher odds of reporting long-term health conditions [8].

This syndemic framing is further supported by evidence demonstrating the mutually reinforcing nature of social inequities, mental health vulnerabilities and occupational exposures in shaping long COVID risks and outcomes. Racialized and marginalized groups, including Hispanic, Asian and Black people, had a higher prevalence of severe psychological distress in 2020, demonstrating the disproportionate psychological burden on racial–ethnic minority groups during these years [9]. Pre-existing mental health disorders are predictors of both severe acute COVID-19 and prolonged neuropsychiatric and somatic manifestations of long COVID [10,11]. In addition, pre-existing mental health disorders have been identified as a risk factor for severe acute COVID-19 infection, as well as the development of persistent symptoms in those with pre-existing mental health disorders. Individuals with long COVID may also face an increased risk of worsening mental health [12]. Finally, it is known that structural inequities shape employment opportunities, and Black and Latino communities are overrepresented in frontline and precarious employment with higher exposure risks. These workers reported elevated pandemic-related stressors [13]. Collectively [13], these intersecting vulnerabilities, such as a racialized identity, mental health history, occupational risk and exposure to systemic inequities, illustrate the syndemic nature of long COVID, whereby co-occurring illnesses interact, and are fueled by social disparities to multiply the burden of disease.

Despite growing recognition of these inequities, there are limited descriptive data on individuals with long COVID and intersecting vulnerabilities, specifically, a racialized identity and pre-existing mental health conditions in Canada. For instance, the literature notes that some Canadian hospitals failed to collect certain race-based COVID data from Black populations, thus further limiting COVID research on this population [14].

The present study aims to descriptively characterize the sociodemographic, symptom, and functional profiles of individuals with long COVID who experience multiple co-occurring social and health-related vulnerabilities, specifically a racialized identity and pre-existing mental health conditions. Grounded in a syndemic framework, the examination of this dual-risk population allows the generation of novel insights into the ways that social determinants of health, structural inequities and disease comorbidities may relate to long COVID experiences, with the potential to inform both healthcare practice and public health strategies that support equitable recovery. From a syndemic perspective, these co-occurring social and health-related vulnerabilities may overlap in ways that compound the burden and experience of long COVID, yet these relationships remain poorly understood.

## 2. Materials and Methods

### 2.1. Study Design and Sample

This study employed a cross-sectional design and represented phase I of a larger mixed-methods project examining the experiences and functional outcomes of individuals from racial and ethnic minority groups in Canada living with Long COVID and pre-existing mental health challenges. Eligibility was determined using self-report screening questions. Participants were eligible if they (1) self-reported symptoms of long COVID as defined by the World Health Organization, (2) self-identified as having a pre-existing mental health challenge, including any DSM-5 disorder or ICD-11 condition, (3) self-identified as belonging to a racialized group, as per visible minority and population groups defined by Statistics Canada, and (4) were adults aged 18–65 years old. Participants were recruited in 2025 using a snowball sampling strategy in collaboration with community organizations supporting racialized communities, including Race and Disability Canada, the Ase Community Foundation for Black Canadians with Disabilities, Independent Living Canada, the Jane-Finch Community Research Partnership, and the University of Toronto Scarborough and Mississauga campuses. Participants completed the survey online via REDCap, a secure electronic data collection tool.

A total of 51 participants met the inclusion criteria and provided informed consent. The study size was determined based on feasibility and exploratory aims, as the first phase of a larger mixed-methods research project. In the absence of population-based sampling frames for racialized individuals in Canada with long COVID and pre-existing mental health conditions, formal a priori sample size calculations were not conducted. As such, recruitment aimed to obtain the maximum achievable sample within the study timeframe through community-based partnerships and snowball sampling. Given the descriptive aim, the achieved sample was considered appropriate for the characterization of symptom, functional and mental health outcomes within a hard-to-reach population, rather than examining causal or mechanistic relationships between variables. Research ethics approval was obtained from the University of Toronto Research Ethics Board (REB), ensuring that study procedures adhered to ethical standards for research involving human participants.

### 2.2. Patient and Public Involvement

Two representatives with lived experience from the Canadian Guidelines for Post COVID-19 Condition (CAN-PCC) were involved in a working group that informed the research. Both contributed as members of the working group alongside researchers and clinicians. Their involvement informed the study focus, design, and interpretation of findings as relevant to individuals living with long COVID.

### 2.3. Study Measures

The 130-item survey collected data regarding sociodemographic variables, long COVID symptoms, disability outcome measures, fatigue, work and social functioning, and malaise using validated instruments.

#### 2.3.1. Sociodemographic Characteristics

The study collected a range of sociodemographic variables, including self-described gender, age, marital status, education level, professional training, primary language, ethnicity (including multiple categories and Aboriginal identity), nationality, and province of birth and residence (including years living in Ontario).

#### 2.3.2. Symptom Burden

Symptom burden was measured using the Post COVID-19 Symptom Checklist (PCSC), which assesses specific post-COVID symptoms across the following domains: respiratory, cardiovascular, gastrointestinal, neurological, psychological, musculoskeletal, and other. Respondents indicate the presence and severity of symptoms using a Likert-style scale, with total scores calculated by summing item ratings so that higher scores indicate a greater symptom burden. The PCSC is an adapted version of the COVID-19 Yorkshire Rehabilitation Scale (C19-YRS), a validated tool designed to assess persistent symptoms following SARS-CoV-2 infection, which has good scaling and high internal consistency (Cronbach’s α = 0.89) [15], supporting the reliability and validity of the adapted PCSC for use in long COVID populations.

#### 2.3.3. Functional Outcomes

Functional outcomes were measured using the Work and Social Functioning Adjustment Scale (WSAS), World Health Organization Disability Assessment Schedule 2.0 (WHO DAS 2.0), and Post-COVID Functional Status Scale (PCFS). The WSAS is a 5-item self-report assessment of the impact of mental health problems on work, home management, social leisure, private leisure, and personal/family relationships. Each item is scored on a 0–8 scale, yielding a total score of 0–40 that reflects low, moderate, or severe impairment. The WSAS has demonstrated strong psychometric properties across clinical populations, including Cronbach’s α ranging from 0.70 to 0.94 and a test–retest reliability of 0.73. These properties have been established in studies of depression and obsessive–compulsive disorder, supporting the scale’s reliability and validity for assessing functional impairment [16]. The WHO DAS 2.0 is a 12-item self-report measure that assesses disability across six functional domains (cognition, mobility, self-care, getting along, life activities, and participation). Items are summed, with a total of up to 48, and higher scores reflect a greater level of disability and functional limitation. In populations with non-acute physical causes of disability, the WHODAS 2.0 has demonstrated strong psychometric properties, including high internal consistency (Cronbach’s α up to 0.96), good test–retest reliability and discriminant validity, and strong correlations with other disability measures [17]. The PCFS measures limitations in daily life (PCFS) due to post-COVID symptoms, classifying the functional status in five categories ranging from no limitations to severe limitations. Higher PCFS scores indicate increasing restrictions in usual activities and participation. Although the PCFS is not yet formally validated, implementing it alongside established outcomes will help generate evidence to assess its usefulness in guiding post-COVID care [18].

#### 2.3.4. Fatigue, Post-Exertional Malaise and Cognitive Outcomes

Fatigue-related impact was measured using the Modified Fatigue Scale, a 21-item self-report evaluation of the impact of fatigue on daily life in the physical, cognitive, and psychosocial domains, with a total score from 0 to 84, where higher scores indicate a greater fatigue impact. The measure has been used in chronic disease populations. For example, it showed high internal consistency in multiple sclerosis (MS) populations for the total score (α = 0.81–0.92) and for the cognitive (α = 0.88–0.95) and physical (α = 0.91–0.92) subscales, while the psychosocial subscale shows somewhat lower consistency (α = 0.65) [19]. Although it has not been extensively studied outside of MS, it has been theorized to be useful for measuring fatigue in long COVID populations [20].

The DePaul Symptom Questionnaire for Post-Exertional Malaise (DSQ-PEM-SF) is a 14-item self-report measure of the frequency and severity of post-exertional malaise (PEM) symptoms, defined as a worsening of symptoms after minor physical or mental exertion. The measure uses five core items rated 0–4 for frequency and severity, with clinically relevant PEM indicated by a score of ≥2 on both scales for any item. The DePaul Symptom Questionnaire (DSQ) is a widely used measure of symptoms in myalgic encephalomyelitis and chronic fatigue syndrome, demonstrating strong test–retest reliability, content validity, and internal consistency.

To reduce the burden, the 14-item short form was used, which captures the most prevalent fatigue symptoms while retaining good discriminative ability [21]. The Perceived Deficits Questionnaire (PDQ-20) was used to assess subjective cognitive impairment, such as memory, attention, and concentration, using the 20-item measure. Total scores were calculated to quantify perceived cognitive impairment, with higher scores indicating a greater perceived deficit. The full-length PDQ demonstrates excellent internal consistency (Cronbach’s α = 0.93) [22].

#### 2.3.5. Mental Health Outcomes

Mental health was measured using the Patient Health Questionnaire-9 (PHQ-9) and Generalized Anxiety Disorder-7 (GAD-7). The PHQ-9 is a 9-item self-report measure assessing the presence and severity of depressive symptoms over the past two weeks. Higher scores indicate a greater depressive symptom severity, with conventional cut-offs for minimal to severe depressive ranges. The PHQ-9 has strong psychometric support, showing high reliability and validity. Internal consistency is robust, with studies across two distinct patient populations reporting Cronbach’s alpha values of 0.86 and 0.89, indicating that the measure consistently captures depressive symptom severity [23,24,25,26]. The GAD-7 is a 7-item self-report measure assessing the presence and severity of generalized anxiety symptoms over the past two weeks. Higher scores indicate greater anxiety severity, with cut-offs for minimal to severe anxiety. The GAD-7 has been shown to be a reliable and valid measure of anxiety, with high internal consistency (α > 0.82) across assessments. It correlates strongly with other anxiety and well-being measures, supporting its convergent validity [27].

#### 2.3.6. Data Analysis

Survey data were exported from REDCap and analyzed using R software (version 4.4.1) for Windows 10. Descriptive statistics were calculated for all variables, including measures of central tendency (mean, median) and dispersion (standard deviation, minimum, maximum). Categorical sociodemographic variables were summarized using counts and percentages. We assessed the normality of continuous variables using visual inspection and summary statistics. As this study was exploratory and symptom and functional measures may be non-normally distributed, both means and medians were reported. Exploratory Spearman correlation analyses were conducted to examine relationships among key mental health, fatigue, cognitive, and functional outcomes. Spearman correlations were selected given that several outcome measures were derived from Likert-type items. Adjustments for multiple comparisons were not performed given the descriptive aims. Correlation analyses were intended to identify preliminary patterns and should be interpreted with caution. The following outcome measures were analyzed: Perceived Deficits Questionnaire (PDQ-20), World Health Organization Disability Assessment Schedule 2.0 (WHO DAS 2.0), Post COVID-19 Symptom Checklist (PCSC) and Post-COVID Functional Status Scale (PCFS), Work and Social Functioning Adjustment Scale (WSAS), Patient Health Questionnaire (PHQ-9), Generalized Anxiety Disorder (GAD-7), Modified Fatigue Impact Scale (MFIS), and DePaul Symptom Questionnaire for Post-Exertional Malaise (DSQ-PEM-SF). For each outcome measure, scoring followed validated procedures and respective instrument guidelines. All nine outcome measures were summarized descriptively to provide an overview of the participant symptom burden, functioning, and mental health. Missing data were handled using a complete-case approach. Participants with incomplete survey responses were excluded from the final analytic sample. Of the 60 participants who initiated the survey, entries identified as duplicates or with substantial missing data were excluded from the final analytic sample, resulting in a final sample of 51 participants. Missing data were assessed descriptively by examining frequency and percentage of missing responses for each variable. Most variables demonstrated low levels of missingness (generally < 5%), although one demographic variable (“years living in Ontario”) had higher missingness (21.6%). No imputation procedures were applied, as analyses were descriptive and focused on fully completed outcome measures.

## 3. Results

### 3.1. Sociodemographic Characteristics

Of the 60 participants who began the study, 51 completed it (completion rate: 85%), while 9 did not complete the study (dropout rate: 15%). A limited comparison of available demographic data between completers and non-completers suggested that non-completers may have been younger on average (mean age: 24 vs. 39 years); however, interpretation is limited by the small number and substantial missing demographic information among incomplete responses. The final sample thus consisted of 51 participants, and their sociodemographic characteristics are presented in Table 1. Table 2 provides a summary of the outcome measure results. Most participants identified as female (64.7%), followed by male (27.5%), non-binary (3.9%), gender queer (2%) or selected not applicable (2%). The majority were married or living in a common-law relationship (62.7%), while 35.3% were single. Educational attainment was generally high, with 41.2% holding a university undergraduate degree and an additional 29.4% reporting a graduate degree. The sample was ethnically diverse, with the largest groups identifying as South Asian (29.4%), White North American (19.6%), Black Caribbean (15.7%), Black African (11.8%), and White European (11.8%), with additional representation from Southeast Asian, East Asian, mixed backgrounds, Aboriginal, Middle Eastern communities, and participants who preferred not to answer. Most participants reported English as their primary language (76.4%), followed by Bengali (5.9%), French (4%), and several other languages, including Hindi, Urdu, Italian, Nepali, Spanish, and Yoruba (each 2%).

### 3.2. Symptom Burden

Participants reported a slight to moderate overall post-COVID symptom burden on the PCSC (M = 2.34, SD = 0.71, 95% CI [2.15, 2.54]). Across symptom domains, mean scores ranged from 2.23 to 2.96. The highest levels were observed for respiratory symptoms (M = 2.96, SD = 1.03), psychological symptoms (M = 2.86, SD = 1.14), and “other” symptoms (M = 2.94, SD = 1.12).

### 3.3. Functional Outcomes

Functional impact was measured using the WSAS, WHO DAS 2.0 and PCFS. Participants reported moderate functional impairment, with mean WSAS scores of 17.35 (SD = 11.04 95% CI [14.32, 20.38]). According to established WSAS interpretive guidelines, scores between 10 and 20 indicate moderate functional impairment. The scores demonstrate moderate impairment in the ability to work, perform home management, engage in social or private leisure activities and maintain close relations [28]. Participants had a mean score of 16.61 (SD = 9.41) on the WHO DAS 2.0, which is scored from 0 to 48, with higher scores indicating greater disability [29]. Furthermore, the greatest impairments on the WHO DAS 2.0 were observed in participation in society (M = 1.82, SD = 0.91) and mobility (M = 1.80, SD = 1.11), with self-care being least affected (M = 0.60, SD = 0.87). The PCFS mean score was 2.20 (SD = 0.92), corresponding to a slight-to-moderate functional status following COVID-19 infection.

### 3.4. Fatigue, Post-Exertional Malaise and Cognitive Outcomes

Fatigue levels were elevated, with a mean Modified Fatigue Impact Scale total score of 43.39 (SD = 19.0, 95% CI [38.17, 48.61]). The measure is scored from 0 to 84, with higher scores reflecting greater fatigue. Though there is no universal “cut off”, in clinical studies measuring illness-related fatigue, scores greater than 38–40 indicate clinically relevant fatigue levels [30]. There was a notable impact of physical (M = 19.96, SD = 8.58) and cognitive fatigue (M = 19.27, SD = 9.65). The DSQ-PEM was used to measure symptom exacerbation following minimal physical or cognitive tasks, and scores were summed to create a total DSQ-PEM score from 0 to 44, with higher scores indicating a greater post-exertional symptom burden. Participants demonstrated a mean score of 22.47 (SD = 10.93, 95% CI [19.47, 25.47]), and there was considerable variability between participants. The most commonly selected symptoms included next-day fatigue or soreness the day after activity and becoming physically tired after minimal exercise. Cognitive symptoms were also measured using the PDQ, which is scored from 0 to 80 for the total score and 0 to 20 for the subscales, with higher scores reflecting greater perceived cognitive difficulties. The mean total score in the sample was 33.43 (SD = 15.51, 95% CI [29.17, 37.69]), with greater perceived challenges reflected in the attention/concentration subscale (M = 9.7, SD = 4.5) and planning/organization subscale (M = 9.4, SD = 4.7).

### 3.5. Mental Health Outcomes

On the GAD-7, participants reported anxiety on the upper end of the mild range, approaching the validated threshold for moderate anxiety (M = 9.27, SD = 5.72), based on established cut-offs of 5, 10, and 15, indicating mild, moderate and severe anxiety symptoms respectively [31]. On the PHQ-9, participants reported depression in the moderate range (M = 11.43, SD = 6.90, 95% CI [9.54, 13.32]), based on established cut-offs of 5, 10, 15, and 20 indicating mild, moderate, moderately severe, and severe depressive symptoms respectively [32].

### 3.6. Overlapping Health and Functional Challenges

Across domains, participants demonstrated co-occurring physical, cognitive, mental health and functional challenges. Elevated fatigue, PEM, cognitive challenges, anxiety and mood disturbance were reported alongside societal participation and mobility limitations. These findings demonstrate overlapping symptom and functional burdens among participants.

### 3.7. Exploratory Correlation Analyses

Exploratory Spearman correlation analyses demonstrated moderate-to-strong associations among mental health, fatigue, cognitive, and functional outcomes (Table 3). Depression and anxiety scores were strongly correlated (ρ = 0.82). Greater fatigue severity was associated with higher disability (ρ = 0.62) and poorer work and social functioning (ρ = 0.67). Perceived cognitive difficulties were also moderately-to-strongly associated with depression (ρ = 0.65), anxiety (ρ = 0.65), and fatigue (ρ = 0.64). Strong associations were additionally observed between disability and work/social functioning measures (ρ = 0.80). Although several moderate to strong associations were observed, the small sample size and variability across outcome measures may contribute to statistical instability and limit the precision of estimates.

## 4. Discussion

This study provides a descriptive characterization of adults in Canada with long COVID who experience intersecting vulnerabilities, specifically a racialized identity and pre-existing mental health conditions. Our findings suggest that this dual-risk population experiences concurrent physical, cognitive, psychological and functional challenges, including a moderate symptom burden, moderate functional impairment, clinically significant fatigue and PEM, moderate perceived cognitive difficulties and elevated mental health symptoms, notably mild anxiety approaching the moderate range and moderate depression. Specifically, respiratory and psychological symptoms were prominent, and functional impairments were most pronounced in societal participation and mobility, while self-care was relatively preserved. This pattern suggests that participants were generally able to maintain personal care and basic activities of daily living (ADLs), but experienced greater difficulties with higher-level functioning, such as instrumental activities of daily living (IADLs), community engagement, and participation in social and community roles.

Our results align with existing evidence suggesting that long COVID is characterized by a heterogeneous symptom profile, with overlapping impacts in the physical, cognitive, psychological, and sensory domains [33]. Elevated fatigue and PEM in our sample are consistent with prior reports linking long COVID to chronic fatigue syndrome-like presentations. Further, cognitive difficulties captured via the PDQ-20 corroborate previous findings of “brain fog” in long COVID populations [34,35]. The novelty of our study lies in its focus on intersecting vulnerabilities. While the existing literature conducted in the US and Europe has identified racial and ethnic disparities in COVID-19 morbidity and mortality [36,37,38], less is known about functional outcomes of long COVID in racialized populations. Our findings suggest that structural inequities and pre-existing mental health challenges may have real consequences for participation in daily life, including home, work, social, leisure and interpersonal domains. Exploratory correlation analyses further support the compounding nature of symptom, mental health, cognitive and functional challenges within the sample. Moderate to strong associations were observed between fatigue, disability, work and social functioning, perceived cognitive difficulties, depression and anxiety. In particular, greater fatigue was associated with higher levels of disability and poorer work and social functioning. Depression and anxiety demonstrated a strong association with perceived cognitive difficulties. The study provides empirical support for the conceptualization of long COVID as a syndemic phenomenon, wherein overlapping health conditions disproportionately affect socially vulnerable populations [39,40].

From a syndemic perspective, several possible mechanisms may be proposed to better understand the symptom burden observed in this population beyond disease processes alone. Yadav et al. (2020) described syndemics as synergistic interactions between biological variables, such as mental health and nutrition, and social–ecological variables, such as urban planning and misogyny, resulting in adverse outcomes [41]. In our sample, pre-existing mental health conditions may interact with biological vulnerabilities associated with long COVID to influence symptom perception, fatigue, and cognitive difficulties [42]. Simultaneously, a racialized identity may shape experiences of illness through healthcare access, differences in symptom recognition or interpretation, and exposure to social and structural stressors, which can compound the overall impact of long COVID. These overlapping disease-related and social challenges may contribute to the functional limitations and reduced participation in IADLs seen in the sample. From a rehabilitation perspective, our findings underscore the need for tailored interventions extending beyond symptom management alone, and also addressing functional restoration and broader social determinants of health. Consistent with prior research, it is often a cluster of symptoms, such as fatigue, PEM, cognitive challenges, and psychological distress, that most strongly impact long-term functioning, social participation, and overall lifestyle [43,44]. In our sample, moderate functional impairments, particularly in societal participation, highlight opportunities for targeted rehabilitation interventions to support return to work and social engagement. Mental health support is critical, as depression and anxiety levels were notable and likely interact with other symptom domains to compound functional challenges.

Several limitations warrant consideration. First, our sample size (*n* = 51) limits the generalizability and feasibility of adequately powered subgroup analyses across demographic and clinical categories. Snowball sampling may also introduce the potential for selection and sampling bias; participants who were more easily recruited, such as those connected to organizations or with reliable internet access, may not fully represent the broader population of racialized individuals with pre-existing mental health conditions. Next, self-report measures are subject to recall and reporting bias. Moreover, the cross-sectional design and descriptive design limit the ability to comment on the mechanisms or interaction pathways through which biological, psychological, and social factors may influence long COVID within a syndemic framework. Accordingly, the proposed syndemic framing and suggested mechanisms should be interpreted as plausible conceptually based explanations for the overlapping burdens in the sample. Finally, although our sample was diverse, certain racialized groups were underrepresented. Longitudinal studies are needed to examine long-term trajectories of symptom persistence, functional recovery, and mental health outcomes. In addition, future research should incorporate inferential statistical analyses to examine relationships among symptom clusters, functional outcomes, and sociodemographic variables, thereby clarifying associations and guiding targeted intervention strategies. Finally, interventions targeting both symptom management and social determinants of health should be evaluated for effectiveness.

## 5. Conclusions

Overall, adults with long COVID who identify as racialized and have pre-existing mental health conditions experience overlapping symptom burden, including cognitive difficulties, fatigue, and functional limitations. These findings progress the understanding of long COVID as a syndemic condition, necessitating the consideration of biological, psychological and social–ecological interfaces.

## Figures and Tables

**Table 1:** Summary of sociodemographic characteristics of participants.

Demographic Characteristics	N	%	Mean	Min.	Max.	SD	95% CI
**Age**			39.69	21.00	64.00	10.09	36.92–42.46
**Gender**							
Female	33.00	64.70					
Male	14.00	27.50					
Non-binary	2.00	3.90					
Gender queer	1.00	2.00					
N/A	1.00	2.00					
**Marital status**							
Married/common law	32.00	62.70					
Single	18.00	35.30					
N/A	1.00	2.00					
**Educational level**							
University undergraduate degree	21.00	41.20					
University graduate degree	15.00	29.40					
College diploma	9.00	17.60					
College certificate	3.00	5.90					
Completed high school	2.00	3.90					
Other	1.00	2.00					
**Ethnicity**							
South Asian	15.00	29.40					
White North American	10.00	19.60					
Black Caribbean	8.00	15.70					
Black African	6.00	11.80					
White European	6.00	11.80					
Southeast Asian	4.00	7.80					
East Asian	3.00	5.90					
Mixed background	3.00	5.90					
Aboriginal	2.00	3.90					
Middle Eastern	1.00	2.00					
Prefer not to answer	3.00	5.90					
**Language**							
English	39.00	76.40					
Bengali	3.00	5.90					
French	2.00	4.00					
Hindi	1.00	2.00					
Urdu	1.00	2.00					
Italian	1.00	2.00					
Nepali	1.00	2.00					
Spanish	1.00	2.00					
Yoruba	1.00	2.00					
N/A	1.00	2.00					

**Table 2:** Summary of outcome measure results.

Survey Measure	Mean	Median	Min.	Max.	SD	95% CI
**Post COVID-19 Symptom Checklist**	2.34	2.23	1.00	4.09	0.71	2.15–2.54
Respiratory symptoms	2.96	3.00	1.00	5.00	1.03	2.68–3.24
Cardiovascular symptoms	2.33	2.25	1.00	5.00	1.05	2.04–2.62
Gastrointestinal symptoms	2.32	2.14	1.00	4.86	0.94	2.06–2.58
Neurological symptoms	2.57	2.45	1.00	5.00	1.09	2.27–2.87
Psychological symptoms	2.86	3.00	1.00	5.00	1.14	2.55–3.17
Musculoskeletal symptoms	2.23	1.75	1.00	5.00	1.23	1.89–2.57
Other symptoms	2.94	3.00	1.00	5.00	1.12	2.63–3.25
**Work and Social Functioning Adjustment Scale**	17.35	17.00	0.00	40.00	11.04	14.32–20.38
**WHO Disability Assessment Schedule 2.0**	16.61	15.00	0.00	36.00	9.41	14.03–19.19
Understanding and communicating	1.39	1.50	0.00	3.00	0.97	1.12–1.66
Getting around	1.80	1.50	0.00	4.00	1.11	1.50–2.10
Self-care	0.60	0.00	0.00	3.50	0.87	0.36–0.84
Getting along with people	1.04	1.00	0.00	3.50	0.94	0.78–1.30
Life activities	1.67	1.50	0.00	3.50	0.97	1.40–1.94
Participation in society	1.82	2.00	0.00	3.50	0.91	1.57–2.07
**Modified Fatigue Impact Scale**	43.39	42.00	0.00	75.00	19.00	38.17–48.61
Physical	19.96	20.00	0.00	36.00	8.58	17.60–22.32
Cognitive	19.27	20.00	0.00	40.00	9.65	16.62–21.92
Psychosocial	4.16	4.00	0.00	8.00	2.22	3.55–4.77
**Generalized Anxiety Disorder**	9.27	9.00	0.00	21.00	5.72	7.70–10.84
**Perceived Deficits Questionnaire**	33.43	32.00	0.00	61.00	15.51	29.17–37.69
Attention/Concentration Subscale	9.67	10.00	0.00	18.00	4.46	8.45–10.89
Retrospective Memory Subscale	7.61	7.00	0.00	16.00	4.19	6.46–8.76
Prospective Memory Subscale	6.76	7.00	0.00	18.00	3.57	5.78–7.74
Planning/Organization Subscale	9.39	8.00	0.00	19.00	4.66	8.11–10.67
**DePaul Symptom Questionnaire for Post-Exertional Malaise**	22.47	21.00	0.00	44.00	10.93	19.47–25.47
Dead, heavy feeling after starting to exercise	1.28	1.33	0.00	3.00	0.83	1.05–1.51
Next day soreness or fatigue after	1.96	1.96	0.00	4.00	1.03	1.68–2.24
Mentally tired after the slightest effort	1.91	2.00	0.00	4.00	1.06	1.62–2.20
Minimum exercise makes you physically tired	2.05	2.00	0.00	4.00	1.15	1.73–2.37
Physically drained or sick after mild activity	1.78	1.75	0.00	4.00	1.12	1.47–2.09
If you were to become exhausted after actively participating in extracurricular activities, sports, or outings with friends, would you recover within an hour or two after the activity ended?	0.44	0.00	0.00	1.00	0.50	0.30–0.58
Do you experience a worsening of your fatigue/energy-related illness after engaging in minimal physical effort?	0.82	1.00	0.00	1.00	0.39	0.71–0.93
Do you experience a worsening of your fatigue/energy-related illness after engaging in minimal mental effort?	0.65	1.00	0.00	1.00	0.48	0.52–0.78
If you feel worse after activities, how long does this last?	1.84	1.50	0.00	5.00	1.53	1.42–2.26
If you do not exercise, is it because exercise makes your symptoms worse?	0.51	1.00	0.00	1.00	0.51	0.37–0.65
**Post-COVID Functional Status Scale**	2.20	2.00	0.00	3.00	0.92	1.95–2.45
**Patient Health Questionnaire**	11.43	10.00	0.00	24.00	6.90	9.54–13.32

**Table 3:** Exploratory Spearman correlations among key outcomes.

Variables	Spearman ρ
PHQ-9 and GAD-7	0.82
MFIS and WSAS	0.67
MFIS and WHODAS 2.0	0.62
PHQ-9 and PDQ-20	0.65
GAD-7 and PDQ-20	0.65
WHODAS 2.0 and WSAS	0.80

## Data Availability

The datasets generated during and/or analyzed during the current study are available from the corresponding author on reasonable request.
